# Older and younger adults’ hindsight bias after positive and negative outcomes

**DOI:** 10.3758/s13421-021-01195-w

**Published:** 2021-06-15

**Authors:** Julia Groß, Ute J. Bayen

**Affiliations:** 1 Heinrich-Heine-Universität Düsseldorf, Institute for Experimental Psychology, 40225 Düsseldorf, Germany; 2 Present Address: Department of Psychology, University of Mannheim, A5, Building C, 68159 Mannheim, Germany

**Keywords:** Hindsight bias, Aging, Memory, Judgment

## Abstract

After learning about facts or outcomes of events, people overestimate in hindsight what they knew in foresight. Prior research has shown that this *hindsight bias* is more pronounced in older than in younger adults. However, this robust finding is based primarily on a specific paradigm that requires generating and recalling numerical judgments to general knowledge questions that deal with emotionally neutral content. As older and younger adults tend to process positive and negative information differently, they might also show differences in hindsight bias after positive and negative outcomes. Furthermore, hindsight bias can manifest itself as a bias in memory for prior given judgments, but also as retrospective impressions of inevitability and foreseeability. Currently, there is no research on age differences in all three manifestations of hindsight bias. In this study, younger (*N* = 46, 18–30 years) and older adults (*N* = 45, 64–90 years) listened to everyday-life scenarios that ended positively or negatively, recalled the expectation they previously held about the outcome (to measure the memory component of hindsight bias), and rated each outcome’s foreseeability and inevitability. Compared with younger adults, older adults recalled their prior expectations as closer to the actual outcomes (i.e., they showed a larger memory component of hindsight bias), and this age difference was more pronounced for negative than for positive outcomes. Inevitability and foreseeability impressions, however, did not differ between the age groups. Thus, there are age differences in hindsight bias after positive and negative outcomes, but only with regard to memory for prior judgments.

**Supplementary Information:**

The online version contains supplementary material available at 10.3758/s13421-021-01195-w.

## Introduction

When we look back on what we knew previously, we are often biased by what we know now. In hindsight, we think that we “knew it all along” (Wood, 1978), we assign higher a priori probabilities to facts or outcomes (Fischhoff, 1975), and we misremember our prior predictions as closer to facts or actual outcomes (e.g., Erdfelder & Buchner, 1998). This cognitive illusion has been termed *hindsight bias* (for reviews, see Blank et al., 2007; Roese & Vohs, 2012) and is pervasive in a variety of everyday situations, such as in political elections, medical diagnoses, or scientific experiments (e.g., Arkes et al., 1981; Blank et al., 2003; Slovic & Fischhoff, 1977). It is also robust across a wide range of materials and different tasks (Pohl, 2007).

In one of the most common types of task to study hindsight bias, the memory paradigm, participants are first asked to provide judgments; for example, numerical judgments to a set of difficult knowledge questions (e.g., *How many letters does the Arabic alphabet contain?*). They then receive the correct answer for some or all of the questions (e.g., *The Arabic alphabet contains 28 letters*). Finally, they are asked to recall their own original judgments. Hindsight bias occurs when recalled judgments are shifted toward presented correct answers. We will refer to the bias measured in the memory paradigm as the *memory component of hindsight bias.*^1^

Research has repeatedly demonstrated that in such numerical-estimation tasks, older adults are more prone to the described memory distortion (e.g., Bayen et al., 2006; Bernstein et al., 2011; Coolin et al., 2015; Groß & Bayen, 2015). In a recent meta-analysis, Groß and Pachur (2019) found that older adults show worse memory for their own original judgments than young adults do, and—as a consequence—must reconstruct their judgments more often. In this reconstruction, older adults are more prone to be biased by the correct answer than younger adults are. This could be due to older adults’ worse memory for their own original judgments (e.g., Groß & Bayen, 2015), and/or to their larger difficulties in inhibiting the correct answer in the reconstruction of original judgments (e.g., Coolin et al., 2015).

In this study, we aimed at extending previous findings on age differences in hindsight bias with regard to two important aspects. Our first main aim was to investigate age differences in hindsight bias in a self-relevant domain with positive and negative outcomes. Our second main aim was to expand our understanding of age differences in hindsight bias by investigating manifestations other than memory distortions—namely, retrospective impressions of foreseeability and inevitability. In the following, we will explain in more detail the importance of these two extensions in research on age differences in hindsight bias.

### Hindsight bias for positive and negative outcomes

The finding that older adults are more prone to hindsight bias than younger adults are is based primarily on studies that investigated memory for numerical judgments in general-knowledge tasks (summarized in Groß & Pachur, 2019).^2^ However, hindsight bias not only occurs after learning facts and figures that are emotionally neutral, but also after emotionally significant outcomes of personal relevance, such as losing a job, missing a sale, or hearing from an old friend (Blank & Peters, 2010; Louie, 1999; Mark & Mellor, 1991; Pezzo & Beckstead, 2008; Tykocinski, 2001). For example, in one of the first studies involving emotionally relevant outcomes, Mark and Mellor (1991) found hindsight bias to be reduced in laid-off manufacturing workers, for whom the layoff was negative, compared with retained workers or community members, for whom the layoff was less consequential. Subsequent studies have found similar decreases in hindsight bias after negative outcomes (Groß et al., 2017; Nestler et al., 2010), but other studies have also found increases in hindsight bias after negative, self-relevant outcomes (e.g., Tykocinski, 2001; Wann et al., 2008). Thus, while the valence of the outcome does affect hindsight bias, this effect is not uniform. As we will explain in more detail in the next section, among the key variables that moderate the effect of outcome valence on hindsight bias in self-relevant domains are the amount of personal controllability of the (positive or negative) outcome (Blank & Peters, 2010), and the hindsight-bias manifestation (component) under consideration (Blank et al., 2008).

To the best of our knowledge, no study has examined age differences in hindsight bias for such event outcomes. There is reason to believe that positive and negative outcomes—that is, outcomes that can be emotionally significant—have a special status in older adults’ cognitive processing. It is thought that older adults shift their goals towards emotional functioning and meaningfulness as their future time perspective becomes less expanded (Carstensen, 2006). In fact, older adults report higher levels of emotional well-being than younger adults (e.g., Carstensen et al., 2011; Kunzmann et al., 2017; Mroczek & Spiro, 2005). Prominent theories of emotional aging postulate that these age-related differences in affective experience can be linked to age-related differences in cognition. Specifically, in comparison with young adults, older adults show a preference for positive over negative material in information processing, including memory (Mather & Carstensen, 2005; Reed et al., 2014). For example, compared with younger adults, older adults tend to recall fewer negative than positive images (Charles et al., 2003), and they recall more positive and less negative affect than do younger adults (Levine & Bluck, 1997; Ready et al., 2007). Age-related differences in cognition may thus be in service of emotion regulation (for a review, see Isaacowitz & Blanchard-Fields, 2012). We assume that this may also show in the processing of positive and negative outcome information in hindsight judgments. Therefore, a first main aim of the current study was to investigate age differences in hindsight bias after positive and negative outcomes.

### Multiple components of hindsight bias

As outlined above, hindsight bias can manifest itself as biased recall of prior given judgments, once the facts or outcomes are known, typically referred to as the memory component of hindsight bias. However, knowing about facts or outcomes of events also affects retrospective judgments of *inevitability* (“It was bound to happen”; Fischhoff, 1975), and retrospective judgments of *foreseeability* (“I knew it all along”; Fischhoff, 1977). To accommodate the different manifestations of hindsight bias, Blank et al. (2008; see also Roese & Vohs, 2012) proposed a multiple-components view on hindsight bias. According to this view, the three components—the memory component of hindsight bias, inevitability impressions, and foreseeability impressions—are independent manifestations of hindsight bias that can occur individually or in combination in reference to an event’s outcome, depending on the requirements of the task. That is, they can be empirically dissociated (e.g., Nestler et al., 2010).

The memory component of hindsight bias is mainly governed by variables that affect memory in general, such as the depth of encoding, the length of the retention interval, and the amount of interference (Erdfelder et al., 2007; Groß & Bayen, 2015; Hell et al., 1988). It has been suggested that this memory component of hindsight bias is a by-product of an adaptive knowledge-updating process (Hoffrage et al., 2000).

Inevitability impressions are a second component of hindsight bias. They occur when one retrospectively thinks “it had to happen,” or when—with outcome knowledge; that is, in hindsight—people assign higher probabilities to outcomes than without outcome knowledge (i.e., in foresight; e.g., Fischhoff, 1975). For example, in a study by Slovic and Fischhoff (1977), participants were asked to rate the probabilities of outcomes of scientific studies (e.g., a female rat showing maternal behavior after having been injected with blood from a mother rat). Participants who had been informed about an alleged true outcome (hindsight group) assigned higher probabilities to these outcomes (and the outcomes’ replicability), compared with participants who had not been informed about a true outcome (foresight group). Inevitability impressions follow from a person’s causal model; therefore, they are stronger when meaningful explanations for an outcome are available, compared with when no explanations are available (Nestler et al., 2010).

Foreseeability impressions are the third component of hindsight bias and refer to the metacognitive belief that one was able to foresee or predict the occurrence of the outcome (“knew-it-all-along” effect). In contrast to inevitability impressions, which refer to the probability of the situation or outcome itself (i.e., Was the outcome inevitable?), foreseeability impressions are judgments about one’s thinking about the outcome (i.e., Did I foresee the outcome?). For example, participants in a study by Hastie et al. (1999) reviewed information about a route of hazardous train tracks. Participants who had learned about the occurrence of a severe accident (hindsight group) indicated that they had foreseen the accident to a greater extent than did those who had not learned about the accident (foresight group). Foreseeability impressions are counter to feelings of surprise (Nestler et al., 2010).

Inevitability impressions and foreseeability impressions are not only different in nature, but they might also serve different psychological functions. Specifically, it has been suggested that individuals may adjust their impressions of inevitability or foreseeability of event outcomes in retrospect in order to regulate negative affect (e.g., Blank et al., 2008; Pezzo & Pezzo, 2007). For example, a person could *increase* the perceived inevitability of a negative outcome (“It was bound to happen anyway”) in order to cope with disappointment. Tykocinski (2001) found that participants who experienced a disappointing outcome (e.g., the failure to secure an attractive deal) retrospectively showed increased impressions of inevitability of the outcome. Such inevitability increases should be helpful primarily when the outcome had been out of one’s personal control (Blank & Peters, 2010).

To avoid regret or self-blame, a person may *decrease* the perceived foreseeability of a negative event outcome (“I didn’t see it coming”; Louie, 1999). Such *un*foreseeability impressions can reduce self-blame for outcomes that had been, at least partly, under the person’s control (Blank & Peters, 2010) and are typically found in contexts with emotional relevance (e.g., Groß et al., 2017; Mark & Mellor, 1991; Nestler et al., 2010). For example, Nestler et al. (2010) found that participants who had experienced a (fictitious) financial loss due to a risky stock purchase rated their loss as less foreseeable in hindsight than in foresight.

Studies that attempted to assess such psychological functions of inevitability and foreseeability impressions, for instance, by examining associations between the hindsight components and affect, have mainly relied on hypothetical scenarios. The results are mixed and inconclusive (e.g., Blank & Peters, 2010; Groß et al., 2017; Tykocinski & Steinberg, 2005). This may indicate that a function of hindsight bias may not be present under all circumstances (discussed by Nestler et al., 2010) or that such a function may be difficult to tap (discussed by Groß et al., 2017).

In sum, it is currently unknown whether age differences in hindsight bias are restricted to the memory component, or whether age differences also emerge for inevitability and foreseeability impressions. A psychological function might be associated with these components of hindsight bias, although empirical evidence is inconclusive. Without knowledge of possible age differences in all three components of hindsight bias, the picture of hindsight bias in older age is incomplete. Therefore, the second main aim of our current study was to fill a research gap by investigating possible age differences in all three components of hindsight bias.

### The present study

To address our two main aims, we examined different manifestations of hindsight bias after positive and negative outcomes of everyday-life scenarios. Healthy older and younger adults listened (via headphones) to a series of everyday-life scenarios that had a positive or a negative outcome. To investigate hindsight bias (i.e., a bias due to outcome knowledge), we had participants rate each hindsight-bias component from both a pre- and a postoutcome perspective.

Apart from our two main aims, we explored two additional questions. First, we included cognitive (recall ability) and motivational (future time perspective, current mood) variables to explore potential associations of these variables with hindsight bias and age differences therein. Second, we explored the relationship of hindsight bias (and age-differences therein) with affective reactions to gauge potential age differences in the assumed psychological functions. To this end, we had older and younger adults rate the affective reaction they thought they would experience after the imagined positive and negative outcomes.

With regard to our first main aim, we expected a stronger memory component of hindsight bias in older compared with younger adults (Groß & Pachur, 2019). In line with previous findings on age differences in emotional information processing (Charles et al., 2003), we expected older adults to (a) hold more positive expectations about the outcomes than younger adults, and (b) recall more scenarios that ended positively rather than negatively, compared with younger adults. With regard to age differences in the memory component of hindsight bias for negative versus positive outcomes, our expectations were less clear-cut. Assuming that memory for expectations followed by negative (vs. positive) outcomes would be worse in older than in younger adults due to an age-related positivity effect in recall, older adults’ hindsight bias should be more pronounced for negative (vs. positive) outcomes, because hindsight bias increases as memory for the original judgment decreases (Groß & Bayen, 2015). However, prior research has also found lower emotional well-being, as indicated by higher levels of depressive symptoms, to be associated with stronger memory hindsight bias for negative (vs. positive) outcomes (Groß et al., 2017). Thus, older adults, whose emotional well-being is superior, might show less pronounced memory hindsight bias for negative outcomes than younger adults. Therefore, age differences in the memory component of hindsight bias for negative versus positive outcomes may depend on the relative influence of cognitive (recall) versus emotional-motivational factors in remembering the initial expectation.

If foreseeability and inevitability impressions have psychological (self-regulation) functions that unfold in reaction to the scenarios, then we should observe pre-to-post outcome *increases* in inevitability and/or *decreases* in foreseeability following negative outcomes, whereas for positive outcomes, pre- to postoutcome shifts should be less pronounced or in the opposite direction. This pattern should be more pronounced in older than in younger adults if hindsight bias has a psychological function relevant to emotional well-being, which is superior in older adults.

## Method

### Participants

The study was approved by the local Ethics Committee. All participants were native speakers of German. The older adults were community residents of the Düsseldorf area and received monetary compensation. They were recruited via newspaper advertisement or personal contact (e.g., at recreational facilities and events for senior citizens). The younger adults were students at Heinrich-Heine-Universität Düsseldorf and received course credit or monetary compensation. They were recruited via social-media platforms and flyers posted on campus.

We recruited participants above the age of 60 (older adults) or below the age of 31 (younger adults). To rule out effects of depressive symptoms on our hindsight measures, we excluded participants with moderate or severe depressive symptoms (i.e., with Beck Depression Inventory, 2nd ed. [BDI-II] scores > 19; Beck et al., 1996). We excluded participants currently under the influence of drugs and those who had consumed more than four alcoholic drinks within 24 hours prior to participating. We further excluded participants with health problems possibly affecting attention and memory (i.e., current neurological or psychiatric illness, unregulated high blood pressure, drug or alcohol abuse, use of sleep-inducing drugs or sedatives, history of stroke, heart attack, brain trauma, brain tumor, emphysema, chemotherapy, and alcohol or drug dependency).

Forty-seven younger and 45 older adults who fulfilled inclusion criteria participated. This sample size is in line with earlier research on the topic (see Groß & Pachur, 2019, Table 2, for an overview). We excluded one younger participant for not following the instructions. Table 1 shows participant characteristics.

**Table 1:** Participant characteristics

		Younger adults (*N* = 46)		Older adults (*N* = 45)		
Age in years	*M* (*SD*)	21.9	(3.0)	73.3	(5.8)	*p* < .05
	range	18–30		64–90		
Gender	female/male	*n* = 31/15		*n* = 29/16		
Education	*M* (*SD*)	15.1	(2.0)	13.7	(3.4)	*p* < .05
Verbal ability	*M* (*SD*)	28.1	(3.8)	32.9	(2.2)	*p* < .05

*Note*. We measured education as total years in high school, vocational school, college, and university. Verbal ability scores were test scores on the 37-point Mehrfach-Wortschatz-Intelligenztest (MWT-B; Lehrl, 1999)

### Materials and measures

#### Scenario descriptions

We used 16 scenario descriptions in the German language (available in the Open Science Framework at https://osf.io/qvhnu/). The scenarios described situations related to health, family, leisure, romance, household, and travel. We used scenarios with outcomes that we considered neither completely uncontrollable nor completely controllable because outcome controllability is a moderator of hindsight bias (Blank & Peters, 2010) that we wanted to control. We adopted 11 scenarios from a previous study with young adults established as neither completely (un-)controllable (for details, see Groß et al., 2017). In addition, we created five new scenarios, for which we assumed neither complete (un-)controllability, and that were similar to the other scenarios in both structure and content domains, such that all scenarios could plausibly take place in the everyday life of both younger and older adults.

We created two descriptions of each scenario, one with a positive outcome and one with a negative outcome. An example of a scenario description (translated from German) is in Appendix 1. (An English translation of all scenarios can be found at: https://osf.io/qvhnu/). In a separate online rating study, the positive and the negative scenario outcomes were rated as comparably likely to occur and comparably realistic (details on this study and its results can be found in the Supplemental Materials).

We divided the 16 scenarios into two subsets of eight scenarios each (Subsets 1 and 2), with similar topics in each subset. For 21 younger and 23 older participants, Subset 1 was presented with a positive and Subset 2 was presented with a negative outcome. For 25 younger and 22 older participants the subset-to-outcome assignment was reversed.

#### Measures at the scenario level

For each scenario, participants provided pre- and postoutcome ratings of imaginability, foreseeability, inevitability, outcome expectation, and affect. All items had been established in previous research (Blank et al., 2008; Blank & Peters, 2010; Groß et al., 2017). Specifically, before learning about the outcome, participants rated the imaginability of the scenario (“I can clearly imagine myself in the described situation”), the foreseeability of the outcome (“I know how the situation will turn out,” “It is difficult to predict how the situation will turn out” [*reverse coded*]), and the inevitability of the outcome (“Under the given circumstances the outcome of the situation is essentially determined,” “Because of the many factors that could influence the outcome of the situation, the outcome is still open” [*reverse coded*]). For each of these items, participants rated their degree of approval on a continuous scale with the anchors *I fully disagree* and *I fully agree* at the extremes; the rating was automatically translated to a number between 0 and 100. As a baseline for our measure of the memory component of hindsight bias, participants rated their expectation about the outcome (“The situation will turn out …”) on a continuous scale ranging from 0 (*negative*) to 100 (*positive*).

After learning about the outcome, participants again rated its foreseeability and inevitability. For this postoutcome measurement of hindsight cognitions, we used the pre-outcome items and worded them in the past tense (e.g., “I knew how the situation would turn out”). To measure the memory component of hindsight bias, participants recalled their prior expectation (“I assumed that the situation would turn out [*negative*–*positive* from 0 to 100]”). Finally, participants rated three items measuring affect (“If I were in the described situation, I’d feel happy/angry/sad”) on a continuous scale ranging from 0 (*I fully disagree*) to 100 (*I fully agree*).

#### Measures at the participant level

We measured mood, future time perspective, verbal ability, recall ability, education, and level of depression at the participant level. We generated three items to measure mood: “At the moment, my mood is [*very bad*–*very good*],” “At the moment, my mood is [*depressed*–*cheerful*],” and “At the moment, I feel [*sad*–*happy*].” Participants provided ratings on a continuous scale, which we translated into values between 0 (maximum negative mood) to 100 (maximum positive mood). We measured future time perspective (FTP) with the German 10-item version of the Future Time Perspective Scale (Carstensen & Lang, 1996) by calculating an average FTP score per participant. We measured verbal ability with the Mehrfachwahl-Wortschatz-Intelligenztest [Multiple-choice vocabulary intelligence test] (MWT-B; Lehrl, 1999). We measured recall ability by asking participants to write down the gist of as many scenarios as they were able to remember (e.g., “dentist appointment,” “cake baking for birthday party”). Answers were coded as “recalled” or “not recalled” by two independent raters, with a high level of agreement (κ = 0.94; disagreement was resolved by the first author based on explanatory comments by the two raters). We measured level of depression with the German version of the BDI-II (Hautzinger et al., 2006), and education as self-reported years of formal schooling (i.e., high school, vocational school, college, university).

### Procedure

Participants were tested individually or in groups of up to four in a university laboratory. After giving informed consent, they completed brief visual and auditory tests and rated their current mood. Via headphones, participants then first listened to all 16 scenario descriptions without the outcomes in randomized order. They were asked to imagine themselves in the situations as well as they could. They were encouraged to close their eyes and imagine additional details, such as sounds and smells. After each scenario, they rated its (preoutcome) imaginability, foreseeability and inevitability, and outcome expectation.

After providing pre-outcome ratings for each scenario, participants again listened to all 16 scenario descriptions in the same randomized order; this time, the positive or negative outcomes were included. Again, participants were asked to imagine themselves in the situations. After each scenario, participants completed (postoutcome) measures of foreseeability and inevitability, recalled their preoutcome expectation (for a measure of the memory component of hindsight bias), and provided affect ratings. The retention interval for each scenario was between 10 and 15 minutes, depending on the time participants took for their ratings. Next, participants filled out the FTP scale, wrote down as many scenarios as they were able to recall (without time limit), and completed the BDI-II, the MWT-B, and a demographics-and-health questionnaire. Finally, participants were debriefed and compensated.

## Results

The data are available in the Open Science Framework (https://osf.io/qvhnu/). We first report preliminary analyses: preoutcome scenario ratings, scenario recall, mood, and FTP for the two age groups. We then report our main results on age differences in the three components of hindsight bias—foreseeability, inevitability, and the memory component of hindsight bias—as a function of scenario outcome valence. We finally report additional results regarding associations of these effects with cognitive and motivational variables, and how affective reactions to the outcomes were related to age and hindsight bias.

Unless noted otherwise, we performed mixed-effect regression analyses in which we accounted for variability between participants and scenarios by including by-participant and by-scenario random intercepts and random slopes (Barr et al., 2013). We report fixed-effects regression coefficients along with their respective *p* values. We implemented all mixed-effect regressions in *R* using *lme4* (Bates et al., 2015), and we obtained *p* values using *lmerTest* (Kuznetsova et al., 2017). We set α at .05.

### Preliminary analyses

Imaginability ratings were higher for older (*M* = 89.0, *SD* = 10.4) than for younger adults (*M* = 77.3, *SD* = 14.9), *b* = 11.70, *p* < .001. For the foreseeability and inevitability components, we averaged the two items for each component and time of measurement to a composite score for each participant and scenario.^3^ Preoutcome foreseeability ratings did not differ between younger (*M* = 61.3; *SD* = 9.8) and older adults (*M* = 59.6, *SD* = 14.6), *p* = .118. The same held true for inevitability ratings (younger: *M* = 51.1, *SD* = 13.1; older: *M* = 52.8, *SD* = 15.5), *p* = .569. Expectations about the outcomes were rather positive than negative (*M* = 69.1, *SD* = 13.0), *p* < .001. Older adults held more positive expectations (*M* = 75.6, *SD* = 13.5) than younger adults (*M* = 62.8, *SD* = 8.8), *p* < .001.

Mean recall was 11.1 of the 16 scenarios (69.3%). Recall was lower in older (*M* = 10.4, *SD* = 2.4) than in younger adults (*M* = 11.8, *SD* = 2.1), *b* = −0.47, *p* = .009, but there was neither a main effect of valence of the scenario outcome on recall, *b* = 0.30, *p* = .161, nor an interaction of age group and valence, *b* = −0.30, *p* = .278 (descriptive statistics are reported in Table 2). Mood was better in older (*M* = 78.3, *SD* = 19.5) than in younger adults (*M* = 60.4, *SD* = 14.8), *b* = 17.80, *p* < .001. As indicated by average FTP scores, older adults perceived their future as less expanded (*M* = 3.6, *SD* = 1.1) than younger adults (*M* = 5.2, *SD* = 0.8), *t*(89) = 7.7, *p* < .001.

**Table 2:** Descriptive statistics for rated affect and number of recalled scenarios

		Younger adults		Older adults	
		*M*	*SD*	*M*	*SD*
Affect rating ^a^ (0–100)	Joy	88.0	(11.1)	84.1	(11.5)
	Anger	73.1	(13.0)	65.4	(20.3)
	Sadness	64.6	(17.0)	60.9	(22.3)
Number of recalled scenarios (0–8)	Positive	6.2	(1.3)	5.0	(1.7)
	Negative	5.9	(1.2)	5.1	(1.6)

*Note*. ^a^ Presented are group means for positive outcomes only (joy), or negative outcomes only (anger, sadness)

### Main results

#### Overall hindsight bias

To measure hindsight bias in the foreseeability and inevitability components, we subtracted each pre-outcome rating from the corresponding postoutcome rating such that a positive value indicates higher foreseeability and inevitability after learning the outcome (i.e., hindsight bias; see also Blank & Nestler, 2006; Nestler et al., 2010; Nestler et al., 2008). To measure the memory-bias component of hindsight bias, we subtracted each preoutcome expectation from the corresponding recalled (post-)outcome expectation and coded these differences such that positive values always indicate a shift in recall towards the presented (positive or negative) outcome.

We observed *reversed* hindsight bias for both foreseeability (*b* = −12.6, *p* < .001) and inevitability (*b* = −8.5, *p* < .001), indicating that across age groups and outcomes, ratings of foreseeability and inevitability *decreased* from the pre-outcome to the postoutcome perspective. We observed a significant hindsight bias for the memory component, *b* = 7.2, *p* < .001, indicating that across age groups and outcomes, participants’ (postoutcome) recall of their expectations shifted toward the presented outcome, compared with their initial (preoutcome) expectations.

#### Effects of age group and outcome valence

**Fig. 1:**
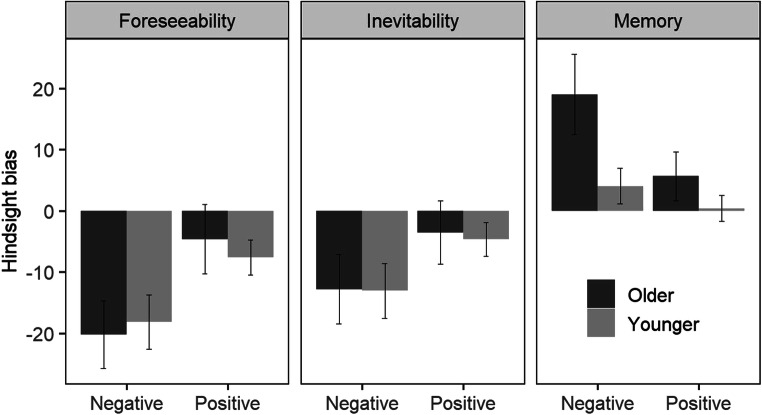
Hindsight bias as a function of age group and outcome valence (negative vs. positive). The bars show mean hindsight-bias scores across participants and scenarios; error bars indicate 95% confidence intervals. For all three components, hindsight bias was calculated as the difference between post-outcome ratings and preoutcome ratings. For the memory component, participants provided ratings on a scale from 0 (*negative*) to 100 (*positive*); therefore, we recoded the post-to-pre-outcome differences such that positive bias scores always represent a shift towards the presented (positive or negative) outcome

To investigate effects of age and outcome valence on hindsight bias, we predicted the hindsight-bias components (foreseeability, inevitability, and memory) from age group, outcome valence, and their interaction (fixed effects). To control for age differences in verbal ability, education, and imaginability, we included these variables as fixed-effect covariates.^4^ Hindsight bias as a function of age group and outcome valence is shown in Fig. 1.

For the foreseeability component of hindsight bias, analyses revealed a main effect of outcome valence, *b* = 10.4, *p* = .029—that is, negative outcomes were associated with a stronger decrease in foreseeability (reversed hindsight bias) than positive outcomes. There was neither an effect of age group, *b* = −0.4, *p* = .936, nor an interaction of age group and outcome valence, *b* = 6.9, *p* = .127, indicating that the foreseeability component of hindsight bias was similar in both age groups.

For the inevitability component of hindsight bias, analyses revealed an effect of outcome valence, *b* = 8.3, *p* = .049—that is, negative outcomes were associated with a stronger decrease in inevitability than positive outcomes. Again, there was neither an effect of age group, *b* = 0.1, *p* = .988, nor an interaction of age group and outcome valence, *b* = 1.5, *p* = .701, indicating that the inevitability component of hindsight bias was similar in both age groups.^5^

For the memory component of hindsight bias, analyses revealed a main effect of age group, *b* = 14.6, *p* < .001, indicating a stronger hindsight bias in older than in younger adults. There was no effect of outcome valence, *b* = −3.7, *p* = .198, but there was an interaction of age group and outcome valence, *b* = −9.2, *p* = .022. Decomposition of the interaction revealed that in older adults, outcome valence affected hindsight bias, *b* = −13.3, *p* < .001, with stronger bias for negative outcomes than for positive outcomes. In younger adults, hindsight bias did not differ between negative and positive outcomes, *b* = −3.6, *p* = .081.^6^

### Additional results

#### Cognitive and motivational influences

In the next step, we investigated whether including cognitive (i.e., recall ability) and motivational (i.e., mood^7^ and FTP) predictors affected age differences in the three hindsight-bias components. That is, as in the first set of analyses, we predicted the hindsight-bias component from age group, outcome valence, and their interaction (fixed effects), with verbal ability, education, and imaginability as fixed-effect covariates. In addition, we included recall ability, mood, and FTP as fixed-effect predictors.

For the foreseeability and inevitability components, respectively, the result pattern remained the same—that is, outcome valence was the only significant predictor (*b* = 10.4, *p* = .029 and *b* = 8.3, *p* = .049, respectively). Neither recall ability, nor mood, nor FTP affected the two hindsight-bias components (all *p*s > .458).

For the memory component of hindsight bias, we conducted separate analyses for younger and older adults, because the effect of outcome valence on the memory component of hindsight bias differed across the two age groups. For younger adults, analyses revealed no significant effects (all *p*s > .073). For older adults, analyses again revealed a significant main effect of outcome valence, *b* = −13.3, *p* = .005, indicating that hindsight bias was stronger for negative than for positive outcomes. In addition, mood and recall ability (but not FTP) were significant predictors: Older adults’ hindsight bias was stronger with better mood, *b* = 0.2, *p* = .007, and stronger with lower recall ability, *b* = −2.5, *p* < .001.

#### Rated affective reactions

Finally, to explore a potential relationship between hindsight bias and affect, we examined the affective reactions that participants rated after positive and negative scenario outcomes (sadness, joy, and anger), and predicted each affective reaction from age group and hindsight-bias component.^8^ Again, we entered verbal ability, education, and imaginability as covariates. Affective reactions did not differ between the age groups (all *p*s > .071; descriptive statistics are in Table 2). Except for joy, which was positively related to the memory component of hindsight bias after positive outcomes, *b* = 0.1, *p* < .001, affective reactions were unrelated to hindsight bias (all *p*s > .058).

## Discussion

This study had two main aims. First, we wanted to investigate age differences in hindsight bias after naturalistic self-relevant positive and negative outcomes. Second, this is the first study to compare all three components of hindsight bias in younger and older adults.

### Age differences in hindsight bias for positive and negative outcomes

In our study, older adults showed stronger overall memory component of hindsight bias than younger adults, replicating prior research on age differences in the memory component of hindsight bias (as summarized by Groß & Pachur, 2019). This is the first study to show that stronger hindsight bias in older adults does not only arise for numerical (or visual) judgments but extends to more self-relevant material.

In the study reported here—as in the studies with numerical judgments—older adults may have been more susceptible to a hindsight bias in the memory component because of their poorer memory for their prior judgments (e.g., Groß & Bayen, 2015; Groß & Pachur, 2019). Note that the design of our study did not allow us to test this hypothesis directly because it did not allow separate measurement of a) recollection of prior expectations from memory and b) measurement of biased reconstruction of prior expectations (for a measurement model that disentangles these processes for numerical judgments, see Erdfelder & Buchner, 1998). However, we found a negative relationship of older adults’ memory for the gist of the presented scenarios (an indicator of general recall ability) with the memory component of hindsight bias. This concurs with earlier reports that age-related differences in episodic memory predicted age-related differences in hindsight bias (Coolin et al., 2015; Groß & Bayen, 2015).

Further, mood was a predictor of the memory component of hindsight bias in older adults (independent of the effect of recall ability). Specifically, better mood was associated with a stronger bias. This finding is consistent with accounts that posit a reliance on heuristic processing (here, anchoring on the actual outcome) in a positive mood (e.g., Huntsinger et al., 2014), and it is also consistent with prior research on the effects of mood on hindsight bias (Groß & Bayen, 2017; Nestler et al., 2010). In younger adults, the memory component of hindsight bias was affected neither by mood nor by recall ability, suggesting that it either depends on other factors or that its variability was too small to be explained by these variables (see Coolin et al., 2015, for a similar result pattern and discussion).

Our study revealed stronger memory component of hindsight bias for negative than for positive outcomes in older, but not younger adults. At first glance, this result may appear to contradict theories that emphasize a positivity bias in older adults’ information processing (Mather & Carstensen, 2005). However, suppose worse memory for one’s prior judgments renders an individual more susceptible to a bias due to outcome knowledge (Groß & Bayen, 2015). In this case, older adults’ stronger memory component of hindsight bias for negative outcomes might indicate that they had worse memory for those outcome expectations that resulted in a negative ending (leading to stronger bias) than those that resulted in a positive ending (leading to a smaller bias). Such a pattern would be in line with the common finding of better memory for positive over negative information in older adults (Charles et al., 2003). However, this account would assume that the bias measured in the memory paradigm was indeed a recollection bias (i.e., outcome knowledge biased the recollection of prior judgments). As explained above, our paradigm did not allow for separate measurements of recollection and reconstruction of one’s prior judgment. Therefore, we do not know if the age-related stronger bias toward negative outcomes was due to biased recollection, biased reconstruction, or other sources. Our measure of recall ability—recall of the gist of the scenarios—showed that there was no age difference in the number of positive versus negative outcomes recalled. Thus, we cannot attribute our finding of age-related differences in the memory component of hindsight bias to an age-related processing bias.

The absence of an age-related processing bias in our study might appear inconsistent with meta-analytic evidence for a positivity effect reported by Reed et al. (2014). However, the authors found that positivity effects were mitigated or absent, when the instructions constrained information processing (e.g., by having participants provide judgments about the stimuli at encoding, or by explicitly asking them to remember information). There were such constraints in our study, which might explain the absence of preferential processing of positive information in older adults. In sum, age-related positivity effects in recall are a possible source of age-related differences in hindsight bias, but there is no direct evidence for them in our study. The remaining methodological challenges should be addressed in future research.

The current results resemble results reported by Groß et al. (2017), who studied hindsight bias in younger adults with wide-ranging levels of depressive symptoms. There, a hindsight bias in the memory component emerged for negative outcomes and was absent for positive outcomes. This difference increased with increasing levels of depressive symptoms. Thus, descriptively, the older adults’ stronger hindsight bias for negative than positive outcomes in the current study mimics the stronger hindsight bias for negative than positive outcomes found in younger adults with depressive symptoms (Groß et al., 2017). However, as we excluded participants with depressive symptoms in the present study, we believe that the two effects—although similar in appearance—result from different mechanisms. First, older adults in the current study had positive expectations regarding the outcomes, whereas individuals with depressive symptoms in Groß et al. had negative expectations. Second, positive mood was associated with stronger bias in older adults in the present study, whereas higher depression levels were associated with stronger bias in individuals with depressive symptoms in Groß et al. Thus, it is likely that different mechanisms resulted in the same observable phenomenon. For future research, we suggest to include control scenarios without outcomes, as these can help to disentangle effects due to the presentation of an outcome from effects that are unrelated to the presentation of an outcome (e.g., general tendency to recall judgments more negatively). For the future, it is an important and challenging endeavor to examine when and how cognitive and emotional factors influence the occurrence and magnitude of hindsight bias and other memory distortions.

### Age differences in hindsight bias are component specific

Our study found diverging effects of aging on the separate components of hindsight bias. While the age groups differed with regard to how they remembered their prior expectations about the outcomes, they did not differ with regard to the subjective foreseeability and inevitability of event outcomes. That is, older and younger adults showed similar pre-to-post outcome shifts in judgments of the outcomes’ foreseeability and inevitability.

For both these components and across age groups and outcomes, we observed *reversed* hindsight bias. That is, after participants had learned the outcomes, they perceived these as *less* foreseeable and *less* inevitable than before. At first glance, this result may seem inconsistent with the large body of research demonstrating robust hindsight bias. However, such reversals of hindsight bias are not uncommon, and prior research has repeatedly demonstrated impressions of *un*foreseeability, specifically after personally relevant and negative outcomes (e.g., Blank et al., 2015; Groß et al., 2017; Mark & Mellor, 1991; Nestler et al., 2010). In line with our predictions, we found the shifts towards *un*foreseeability to be more pronounced for negative outcomes, and small or absent for positive outcomes—a result supportive of a self-protective function (“I didn’t see that coming!”). Alternatively, rather than indicating a psychological function, unforeseeability of negative outcomes may be a simple reflection of the participants’ generally optimistic beliefs (Sharot, 2011; Weinstein, 1980), as expressed in their positive expectations.

Our finding of a *decrease* in inevitability impressions contradicts accounts that propose a coping function of *increases* in inevitability after negative outcomes (e.g., Tykocinski, 2001; but see Groß et al., 2017, for a similar finding). Our finding likely implicates that it was difficult for participants to find explanations for the outcomes. Causal explanations are a major driving force behind inevitability impressions (Nestler et al., 2008). In contrast to other studies that also investigated foreseeability and inevitability within the same paradigm (e.g., Blank & Peters, 2010), we did not provide explanations or additional information that could help make sense of the outcomes. Finding explanations may have been particularly difficult for negative outcomes, as suggested by a decrease in inevitability that was more pronounced for negative than positive outcomes. Again, rather than indicating a specific psychological function, this finding may simply reflect generally positive schemata in our healthy participants. Note that this finding also replicates our prior study, which showed decreases in both foreseeability and inevitability in healthy younger adults for scenarios without additional explanatory information (Groß et al., 2017).

In sum, the similar decrease in foreseeability and inevitability—in particular for negative outcomes—is thus not (entirely) in line with the possibility that these hindsight impressions can regulate negative affect (i.e., promote coping and aid self-protection). The most likely explanation is that our experimental set-up did not cause strong imagined affect that required regulation. We discuss this point in more detail below. Instead, the similar shifts in foreseeability and inevitability point toward cognitive explanations (see also Blank et al., 2008; Blank & Nestler, 2006; Groß et al., 2017). Specifically, metacognition and causal explanations have been proposed to underlie the foreseeability and inevitability components of hindsight bias, respectively (Blank et al., 2008).

As we found no age difference in foreseeability and inevitability impressions, one possible conclusion from our study could be that underlying processes did not differ between older and younger adults. However, it is also possible that age differences in underlying processes result in similar observable hindsight judgments (for an example, see Bayen et al., 2006, Experiment 3; and see our discussion below). Our study was not designed to investigate metacognition and causal explanations as processes underlying hindsight judgments; thus, future research should attempt to clarify their role in hindsight judgments in younger and older adults.

In contrast to our findings on the memory component of hindsight bias, we found no support for older adults’ differential processing of positive versus negative outcomes in the foreseeability and inevitability components of hindsight bias. How can this finding be explained? For the measurement of the memory component of hindsight bias, participants are asked to recall their preoutcome expectations, whereas for the measurement of foreseeability bias and inevitability bias, they provide new postoutcome judgments. That is, for the latter two, there is no demand on episodic memory. Our study therefore adds to the evidence that age differences in cognitive factors (specifically, recall ability) are an important driving force behind age differences in the memory component of hindsight bias (see also Groß & Bayen, 2015).

There was no effect of future time perspective in any of our analyses. Age differences in future time perspective thus cannot explain age differences in hindsight bias in this study. This finding resonates with research that questions future time perspective as a key explanatory construct behind emotional well-being in older adulthood (e.g., Grühn et al., 2015). Future research should attempt to pinpoint the conditions under which future time perspective is or is not predictive of behavior in experimental tasks.

### Hindsight bias and affective self-regulation: A topic for future research

Like prior research, the present study did not show a strong relationship between hindsight bias and affect. Our scenarios were developed to represent the ups and downs of daily life of older and younger adults, but they were merely hypothetical. Future research should investigate possible associations between hindsight bias and affect by relying on genuine positive and negative experiences. These might promote the use of hindsight judgments to regulate affect. In addition, it would be important to clarify the causal direction between hindsight bias and affect. Specifically, we found that the memory component of hindsight bias for positive outcomes was associated with higher levels of joy. While hindsight bias may lead to increased levels of joy, joy may also increase the probability of positively biased recall (e.g., Eich et al., 1994). By manipulating the strength of hindsight bias, it might be possible to disambiguate this association in future research.

### Conclusions and outlook

In this study, we found age differences in hindsight bias in a paradigm with positive and negative event outcomes. Specifically, we found a stronger memory component of hindsight bias in older than in younger adults, which was particularly pronounced for negative outcomes. According to our results, age differences in both cognition and emotion drive this age difference. Our study provides a comprehensive view on hindsight bias in older adulthood by showing that age differences are component specific. We thus extended prior research on age differences in hindsight with regard to two important aspects: the judgment domain and hindsight-bias components. Among the next steps towards a better understanding of this result we propose to investigate the underlying mechanisms of the memory component of hindsight bias for outcomes of emotional significance (e.g., biases in recall, mood), the underlying mechanisms of foreseeability and inevitability impressions (e.g., metacognition, causal explanations), and their relative contributions to hindsight bias in younger and older adults. Additionally, it is important to track down potential self-regulatory functions of hindsight bias with appropriate procedures.

### Supplementary Information

ESM 1 (DOCX 22 kb)

## Appendix 1

Scenario description with positive and negative outcome (translated from German)

*Imagine that you have been attending a weekly dancing class for years. You arrive at the hall and change into your dancing shoes. You say hello to some of your dancing fellows and chat with them about the past week. Then the class starts.*

*The training is going well for you. You and your dancing partner are dancing in step during the whole class. You even succeed in performing a spin, which you had never before managed to do. Afterwards, you are very pleased and drive home in a cheerful mood.* (positive outcome)

*The training is not going well for you. You seem to lack concentration, and you are not dancing in step. You even twist your ankle. Your ankle hurts, and your dancing partner is annoyed at you.* (negative outcome)

## Footnotes

1 It is important to clarify that with the term *memory component of hindsight bias*, we refer to the hindsight bias that is measured in the memory paradigm. The term does not refer to possible processes that underlie this bias (which could be impaired memory or biased reconstruction; e.g., Erdfelder & Buchner, 1998).

2 The only exception we are aware of is the study by Bernstein et al. (2011), who found older adults to be more prone to hindsight bias than young adults are in a task that required visual identification of (emotionally neutral) objects.

3 Mean Cronbach’s α for foreseeability items was 0.68 (pre) and 0.67 (post), and for inevitability items 0.68 (pre) and 0.74 (post).

4 Education affected inevitability and memory hindsight-bias measures such that fewer years of education were associated with stronger hindsight bias. Yet, in all analyses, predictor effects were identical both with and without inclusion of education as a covariate. In none of the analyses was there an effect of verbal ability or imaginability.

5 An anonymous reviewer pointed out that the outcome is clearly specified when participants provide postoutcome foreseeability and inevitability ratings, but vague when they provide preoutcome ratings. To rule out that this might be problematic for the interpretation of the pre-to-post outcome difference measures, we also performed analyses on foreseeability and inevitability with postoutcome ratings only. For both components, we observed the identical result patterns; that is, we found the same effects of valence, and no interaction of age group and valence.

6 Correlations between the hindsight-bias components are reported in the Supplemental Materials.

7 The three mood items were averaged into a composite score for each participant (mean Cronbach’s α = .93).

8 We centered each hindsight-bias measure by subtracting the hindsight-bias scenario mean from each individual hindsight-bias score.
