# Supplementary material for: Older and younger adults’ hindsight bias after positive and negative outcomes
Source: Mem Cognit. 2021 Jun 15;50(1):16–28. doi: 10.3758/s13421-021-01195-w (PMC8763826; doi:10.3758/s13421-021-01195-w)
Supplement: Supplementary file 1 — (DOCX 22 kb) [file 13421_2021_1195_MOESM1_ESM.docx]

**Supplemental materials**

Ratings of Scenario Characteristics

We performed an online rating study to test if positive and negative outcomes were perceived as comparably likely to occur and comparably realistic – two characteristics that could potentially lead to differences in foreseeability (and other) ratings. *N* = 47 young-adult participants (*M*_age_ = 21.0, *SD* = 3.1) read the 16 scenario descriptions, 8 with a positive and 8 with a negative outcome (*n* = 24 with scenario-outcome combination A, and *n* = 23 with scenario-outcome combination B, randomly assigned). Participants were asked to judge how likely and how realistic the outcome was (to make instructions as clear as possible, they also judged how realistic the whole scenario was). Participants used rating scales from 1 (unlikely/unrealistic) to 7 (very likely/very realistic). Descriptive statistics are in Table S1. To test differences between positive and negative outcomes, we took the same statistical approach as in the main experiment; that is, we performed mixed-effects regression analyses to control for various dependencies in the data (participants, scenarios). The differences between ratings for positive versus negative outcomes were not significant (*p*s > .290). Thus, there was no indication that valence of the outcomes was confounded with these two characteristics. Differences in our dependent variables were thus most likely due to the manipulation of valence.

Table S1

*Descriptive Statistics for Likelihood and Realism Ratings of Scenarios*

|  |  | Positive | |  | Negative | |
| --- | --- | --- | --- | --- | --- | --- |
|  |  | *M* | *SD* |  | *M* | *SD* |
| Realistic | Whole Scenario | 5.2 | (0.9) |  | 4.9 | (0.7) |
|  | Outcome | 5.4 | (0.8) |  | 5.0 | (0.8) |
| Likely | Outcome | 4.7 | (0.8) |  | 4.3 | (0.7) |

*Note*. Responses were given on a Likert-scale ranging from 1 (unlikely/unrealistic) to 7 (very likely/very realistic).

Table S2

*Correlations Between Hindsight-bias Measures*

|  | Measure | 1 | 2 | 3 |  | 4 | 5 | 6 |
| --- | --- | --- | --- | --- | --- | --- | --- | --- |
| Positive outcomes | |  |  |  |  |  |  |  |
| 1 | Foreseeability HB | — | .64* | .36* |  | .14 | .23 | .10 |
| 2 | Inevitability HB | .33* | — | .37* |  | .01 | .33* | .07 |
| 3 | Memory HB | .30* | .16 | — |  | .19 | .29* | .16 |
| Negative outcomes | |  |  |  |  |  |  |  |
| 4 | Foreseeability HB | .17 | .24 | .13 |  | — | .75* | .04 |
| 5 | Inevitability HB | .10 | .44* | .06 |  | .75* | — | .26 |
| 6 | Memory HB | .10 | .02 | .28 |  | .20 | .18 | — |

*Note.* HB = Hindsight Bias; Pearson’s correlation coefficients are shown separately for older adults (above diagonal) and young adults (below diagonal).

* *p* < .05
